# Association of NKX2‐5, GATA4, and TBX5 polymorphisms with congenital heart disease in Egyptian children

**DOI:** 10.1002/mgg3.612

**Published:** 2019-03-04

**Authors:** Eman G. Behiry, Mahmoud A. Al‐Azzouny, Dina Sabry, Ola G. Behairy, Nessrine E. Salem

**Affiliations:** ^1^ Clinical and Chemical Pathology Department, Benha Faculty of Medicine Benha University Benha Egypt; ^2^ Biochemistry Department, Cairo Faculty of Medicine Cairo University Cairo Egypt; ^3^ Pediatrics Department, Benha Faculty of Medicine Benha University Benha Egypt; ^4^ Histology Department, Benha Faculty of Medicine Benha University Benha Egypt

**Keywords:** Congenital heart disease, *GATA4*, *NKX2‐5*, *TBX5*

## Abstract

**Background:**

Several genes encoding transcription factors are known to be the primary cause of congenital heart disease. *NKX2‐5* and *GATA4* were the first congenital heart disease–causing genes identified by linkage analysis. This study designed to study the association of five single–nucleotide variants of *NKX2‐5, GATA4*, *and TBX5* genes with sporadic nonsyndromic cases of a congenital cardiac septal defect in Egyptian children.

**Methods:**

Venous blood samples from 150 congenital heart disease children (including a ventricular septal defect, atrial septal defect, tetralogy of Fallot, and patent ductus arteriosus) and 90 apparently healthy of matched age and sex were studied by polymerase chain reaction followed by direct sequencing in order to study two single–nucleotide variants of *NKX2‐5* (rs2277923, rs28936670), two single–nucleotide variants *of GATA4* (rs368418329, rs56166237) and one single–nucleotide variant *TBX5 (*rs6489957). The distribution of genotype and allele frequency in the congenital heart diseases (CHD) group and control group were analyzed.

**Results:**

We found different genotype frequencies of the two variants of *NKX2‐5*, as CT genotype of rs2277923 was present in 58% and 36% in cases and control respectively, and TT genotype present in 6% of the cases. Also regarding missense variant rs28936670, heterozygous AG presented in 82% of the cases. Also, we observed a five prime UTR variant rs368418329, GT (42% of the cases) and GG (46% of the cases) genotypes showed the most frequent presentation in cases. While regarding a synonymous variant rs56166237, GT and GG were the most presented in cases (41.4%, 56% respectively) in contrast to control group (20%, 1.7% respectively). Also, a synonymous variant in *TBX5*, the distribution of genotype frequency was significantly different between the CHD group and control group. CT genotype of *TBX5* ‐rs6489957 was found in 12 ASD, 24 VSD, six PDA, three aortic coarctation and nine fallot that represent 42% of the cases.

**Conclusions:**

Significantly higher frequency of different allelle of five variants was observed in cases when compared to the control group, with significant risky effect for the development of septal defect. In addition to two polymorphisms of *NKX2‐5* (rs2277923, rs28936670) *variant* in the cardiac septal defect, two variants in *GATA4* (rs368418329, rs56166237) and one variant in *TBX5* (rs6489957) seem to have a role in the pathogenesis of congenital heart disease.

## 1 BACKGROUND

Congenital heart diseases (CHD) could be a major cause of morbidity and mortality in children, a previous study revealed that CHD occurred in 80.8% as a solitary lesion, and in 19.2% of the cases they were combined with other cardiac lesions (Bassili et al., 2000). The prevalence increased over time in total CHD birth, from 0.6 to 9.1 per 1,000 live births from 1930 to 1934. Stabilization occurred all over the last 15 years, reaching 1.35 million newborns with CHD every year (van der Linde et al., 2011).

Several studies have been made to identify genes that could be responsible for syndromic and nonsyndromic forms of CHD, by identifying the human gene mutations associated, it has been estimated that *NKX2‐5* (OMIM: 600,584)*,* mutations account for at least 4% of fallot's tetralogy cases. Also authors found that nonsyndromic cardiac septal defects have been linked to mutations in *GATA4* (OMIM: 600,576) (McDermott, Basson, & Hatcher, 2006). Nineteen mutations in *GATA4* have been studied in patients with atrial septal defect (ASD), ventricular septal defect (VSD), and Fallot's tetralogy (Mattapally, Nizamuddin, Murthy, Thangaraj, and Banerjee (2015)).

Mutations in genes known to be resposible for cardiac development such as *NKX2‐5, TBX20, GATA4, GATA6,* and *MYH6* have been studied in families with isolated, nonsyndromic cardioavascular deformities, these genes are resposible for cardiac development in animal models. *GATA4, NKX2‐5*, and *TBX5* (the gene causing Holt‐Oram syndrome) may function in a complex to regulate a group of genes required for formation of cardiac septa (Ware & Jefferies, 2012).

*TBX5* changes cause both skeletal and cardiovascular phenotypes. It has been revealed that major cardiovascular yet milder skeletal abnormalities are because of missense alterations at the amino terminal of the DNA binding domain (Basson et al., 1999). Human *NKX2‐5* is imparted in the cardiovascular primordia from day 7 and is an early sign of both embryonic heart fields development (Stanley et al., 2004). Though alterations in *NKX2‐5* are linked to an extensive variety of CHDs and thyroid dysgenesis, *NKX2‐5* codes for a homeodomain–containing transcription factor with an important role in heart development, and mutations affecting this gene in individuals with congenital heart disease were reported (Dentice et al., 2006).

*NKX2‐5* has a role in nearly almost all phases of heart development, including the regulation of number of cardiac progenitor cells, conduction system development, valve formation, and septation. Interestingly, *NKX2‐5* acts in combination with other transcription factors that are highly‐conserved to organize cardiogenesis. Genome–wide expression analysis has begun to investigate *NKX2‐5* dependent genes (Elliott, Kirk, Schaft, & Harvey, 2010).

Previous authors have identified an unknown mutation in the *TBX5* (OMIM: 601,620) leading to an amino acid change at position 85 from proline to threonine. The mutation had dramatically reduced biological activity that lead to clinical HOS phenotype (Dreßen et al., 2016).

This study planned to study two single–nucleotide variants of *NKX2‐5* (rs2277923, rs28936670), two single–nucleotide variants *of GATA4* (rs368418329, rs56166237) and one single–nucleotide variant *TBX5 (*rs6489957) in sporadic nonsyndromic CHD cases in Egyptian children with congenital cardiac septal deformity from Qalubeya.

## 2 METHODS

This research was accepted by the Research Ethics Committee of Benha University according to the “World Medical Association Declaration of Helsinki 1964” (Idänpään‐Heikkilä, 2001). Written informed consents were obtained from the Guardians of all participants.

### 2.1 Subjects

In this case‐controlled study, we recruited 150 unrelated affected children with non‐syndromic isolated and non‐isolated cardiac septal defects from Benha University Hospital, they were 84 females and 66 males with age ranged from days to 4 years with a mean age (7.8 ± 2.2) months during the period from March 2017 to May 2018. Ninety healthy children with matched age and sex and without a family history of cardiac diseases served as a control group. Patients were diagnosed according to ESC Guidelines for congenital heart disease (Baumgartner et al., 2010). Syndromic CHD cases diagnosed by clinical examination and karyotyping or cases with congenital heart malformations without septation defects were excluded.

### 2.2 Methods

#### 2.2.1 Clinical evaluation

For each case, three‐generation pedigree constructions, Complete history of patients obtained from their parents or medical records and detailed clinical examination were conducted for all participants. Echocardiography, electrocardiogram and Plain chest X‐rays were carried out in all cases.

#### 2.2.2 Cytogenetic studies

Conventional cytogenetic analysis using GTG banding technique and Fluorescence In Situ Hybridization was done for all participants to exclude chromosomal aberration syndromes.

#### 2.2.3 Molecular studies

All subjects were genotyped for five single–nucleotide variants of *NKX2‐5* (rs2277923, rs28936670), *GATA4* (rs368418329, rs56166237) and *TBX5* (rs6489957) using direct Sanger sequencing technique. Five SNPs were selected from the dbSNP database (https://www.ncbi.nlm.nih.gov/projects/SNP/) in the following steps:

##### DNA extraction

Three millilitres of venous blood samples was collected under a complete aseptic condition in vacutainers containing Na2EDTA as an anticoagulant. Genomic DNA was extracted from the peripheral blood sample according to the procedures of the DNA isolation kit iNtRON G‐spin Total DNA extraction kit, catalogue number 17045, Korea (https://www.intronbio.com/eg/).

##### Genotyping by sanger sequencing method

###### Genotypic analysis

The analysis was performed by conventional polymerase chain reaction (PCR) followed by DNA sequencing to examine two single nucleotide polymorphisms in *NKX2‐5*, in comparison with sequence number NM_004387.3 for genome, first synonymous variant rs2277923 (NM_004387.3:c.63T/C) and second is missense variant rs28936670 (NM_004387.3:c.73 G/A), and this study examined two single nucleotide polymorphisms i*n GATA4* according to sequence number NM_002052.4,,first 5 prime UTR variant rs368418329 (NM_002052.4:c.‐294G/T) and synonymous variant rs56166237 (NM_002052.4:c.99 G/T), and synonymous variant rs6489957 in *TBX5* according to sequence number NM_000192.3 (NM_000192.3:c.1281C/T).

Polymerase chain reaction (PCR) was carried out using a total of ten PCR primers that were designed using primer design software (Premier Biosoft Inc., Palo Alto). The amplification was performed in a reaction mixture of 50 μl containing approximately 2 µl genomic DNA, 25 µl PCR Master mix (2x) containing Taq DNA polymerase, dNTPs, 10Mm buffer containing 2mM MgCl2 (iNtRON‐Korea), 10 picomole from each primer and 22 µl Distilled Water (Table 1).

**Table 1:** Primers sequence of the primers used for the polymerase chain reaction (PCR) Amplification of five single–nucleotide variants

SNP	Site	Forward primer	Reverse primer	Size
*NKX2‐5*‐ (rs2277923) c.63C/T	Exon‐1	tgacacgaaactgctcatcg	gtaggcctctggcttgaagg	416 bp
*NKX2‐5*‐ *(*rs28936670*)* c.73G/A	Exon‐1	ctggcgctgtgagactgg	agtttcttggggacgaaagc	422 bp
*GATA4‐*(rs368418329) c.‐294G/T	5‐prime UTR variant	gtgggttctgaaagctctgg	cctcggtgtcctctctctcc	497 bp
*GATA4* (rs56166237) c.99G/T	Exon‐2	cacgcatattatcgttgttgc	gccctggaggtaggacagg	267 bp
*TBX5*‐(rs6489957) c.1281C/T		ttggccaaataactgtctcc	gctggaacattccctctcc	465 bp

The amplified products were subjected to electrophoresis on a 2% agarose gel containing ethidium bromide and visualized using ultraviolet (UV) light trans‐illumination. The PCR products were purified with wash steps and then the DNA was eluted in a low salt buffer. DNA was allowed to adsorb specifically to the silica membrane of a MEGA quick spin column and then sequenced directly by the ABI3730XL sequencer in LGC genomic GmbH, 12,459 Berlin/Germany (WWW.igcgroup.com).

### 2.3 Statistical analysis

The data were organized using SPSS version 16 soft ware (SPSS Inc, Chicago). Quantitative data were designed in the form of Mean and standard deviation, the significance of difference was tested using: ‐Student's *t‐*test and Mann–Whitney Test (*U* test) that was used to compare the mean of two groups of quantitative data. Categorical data were presented as number and percentages. Odds ratios (ORs) and the corresponding 95% CI were calculated. Regression analysis with the adjusted Odds Ratios was used to detect the significant predictors of congenital heart disease. *p* < 0.05 was considered significant.

## 3 RESULTS

The present study was conducted on 150 congenital heart disease (CHD) cases, their mean age was 7.8 ± 2.2 months; there were 66 males (44%) and 84 females (56%). In addition to 90 healthy control subjects of matched age and gender. Thirty three congenital heart disease children (22%) had positive first degree consanguinity, while nine control subjects (10%) had positive first degree consanguinity.

Applying Hardy Weinberg equation revealed that all studied single–nucleotide polymorphisms (SNPs) in control as well as in cases; groups were in HW equilibrium. Clinical and cardiac findings are summarized (Table 2). Five single–nucleotide polymorphisms were reported: a synonymous variant rs2277923, and a missense variant rs28936670 in *NKX2‐5* and 5 prime UTR variant rs368418329 and a synonymous variant rs56166237 in *GATA4* , and a synonymous variant rs6489957 in *TBX5*.

**Table 2:** Clinical presentation and cardiac findings of all studied cases

	Cases *N* = 150	
	*N*	%
LV enlargement	3	2
RV enlargement	60	40
Biventricular enlargement	6	4
ASD	27	18
VSD	51	34
PDA	9	6
TGA	12	8
Aortic coarctation	6	4
Epstein anomaly	6	4
Fallot tetralogy	12	8
complete AV canal	6	4
atrial fibrillation	9	6
heart block	9	6
LV strain pattern	3	2
p pulmonal,rt axis deviation	33	22
sinus tachcardia	15	10
RV stress pattern	6	4
	Mean	*SD*
EF	57	12

Note

ASD: atrial septal defect; VSD: ventricular septal defect; PDA: patent ductus arteriosus; TGA: transposition of great arteries; EF: ejection fraction.

**Figure 1:**
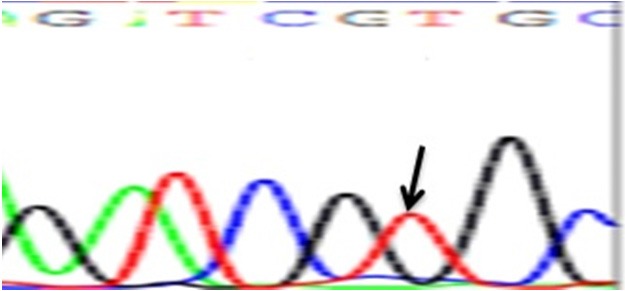
Electrophoretograms showing *NKX2‐5* polymorphism rs2277923 (NM_004387.3:c.63 C/T) identified in homozygous samples (TT)

There were significant different genotype frequencies in cases with CHD than control group regarding five variants (Table 3). Regarding synonymous variant rs2277923 in *NKX2‐5*, CT and TT genotypes were presented more frequent in CHD cases (15 ASD, 24 VSD, six PDA, six aortic coarctation and nine Fallot's tetralogy) than control group, as CT genotype was present in 58% and 36% in cases and control respectively. TT genotype was present in 6% of the cases (three ASD patients) and not present in control (Figure 1), also regarding missense variant rs28936670, heterozygous AG shows that it was most frequently presented in 82% of the cases (27 ASD, 44 VSD, nine PDA and six Fallot's tetralogy) than the control group (36.7%), while the AA genotype was present in 8% of the CHD cases (nine VSD and six Fallot's tetralogy) (Table 4).

**Table 3:** Comparison of studied five SNPs genotypes and alleles between cases and control groups

		Control *N* = 90		Cases *N* = 150					
		*N*	%	*N*	%	*p*	OR	95% CI	
*NKX2‐5* (rs2277923)	*CC*	58	64	54	36	1 (Reference)			
	*CT*	32	36	87	58	0.00	0.3	0.16	0.5
	*TT*	0	0	9	6	0.3	Not applicable		
	*C*	148	82	195	65	1 (Reference)			
	*T*	32	18	105	35	0.002	0.28	0.12	0.6
*NKX2–5*‐ (rs28936670)	*AA*	57	63.3	27	8	1 (Reference)			
	*AG*	33	36.7	123	82	0.00	0.11	0.06	0.2
	*GG*	0	0	0	0		Not applicable		
	*A*	147	81.6	177	59	1 (Reference)			
	*G*	33	18.4	123	41	0.00	0.24	0.109	0.53
*GATA4* (rs368418329)	*GT*	45	50	63	42	1 (Reference)			
	*TT*	33	36.6	18	12	0.004	3.23	1.4	7.2
	*GG*	12	13.4	69	46	0.00	0.224	0.104	0.483
	*G*	111	62	99	33	1 (Reference)			
	*T*	69	38	201	67	0.00	6.7	3.5	12.7
*GATA4*‐ (rs56166237)	*TT*	39	78.3	4	2.6	1 (Reference)			
	*GT*	33	20.0	62	41.4	0.00	0.129	0.048	0.34
	*GG*	18	1.7	84	56	0.00	0.071	0.25	0.2
	*T*	111	62	70	23	1 (Reference)			
	*G*	69	38	230	77	0.001	2.6	1.4	4.7
*TBX5*‐(rs6489957)	*TT*	47	52.2	0	0	1 (Reference)			
	*CT*	41	45.5	63	42	0.00	0.03	0.009	0.109
	*CC*	2	0.3	87	58	0.00	0.003	0.001	0.017
	*T*	135	75	63	21	1 (Reference)			
	*C*	45	25	237	79	0.00	0.187	0.09	0.37

Note

R: reference; OR: odds ratio; CI: confidence interval.

Logistic regression test was used.

**Table 4:** Comparison of cardiac data according to *NKX2‐5* (rs2277923 and rs28936670) in all studied cases

NKX 2–5 (rs2277923) in all studied cases								
	CC *N* = 54		CT *N* = 87		TT *N* = 9		P1*	*P2#*
	*N*	%	*N*	%	*N*	%		
ASD	12	22	12	14*	3	33.3*#	0.00	0.00
VSD	27	50	24	28.5*	0	0*#	0.00	0.00
PDA	3	5.5	6	6.8	0	0*#	0.00	0.00
Aortic coarctation	0	0	6	6.8*	0	0*#*	0.00	0.00
Fallot tetralogy	3	5.5	9	10.3*	0	0*#	0.00	0.00
	Mean	*SD*	Mean	*SD*	Mean	*SD*		
EF	56	0.14	57	0.11	55	0.1	>0.05	>0.05

NKX 2–5 (rs28936670) in all studied cases					
	AA *N* = 27		AG *N* = 123		*P*
	*N*	%	*N*	%	
					
ASD	0	0	27	22	0.00
VSD	9	33	44	35.7	>0.05
PDA	0	0	9	7.3	0.00
Aortic coarctation	0	0	6	4.8	0.001
Fallot tetralogy	6	22	6	4.8	0.00
	Mean	*SD*	Mean	*SD*	
EF	57.2	10.1	60.9	8.4	>0.05

Note

Numerical data are expressed in mean ± *SD*, compared between two groups by *t*‐test; and between more than two groups by ANOVA.Categorical data are expressed in frequency (percentage) and compared by chi square or Fisher exact tests.

GenBank reference sequence:Variant rs2277923 in *NKX2‐5* (NM_004387.3:c.63T/C) and variant rs28936670 in *NKX2‐5* (NM_004387.3:c.73 G/A).

**Figure 2:**
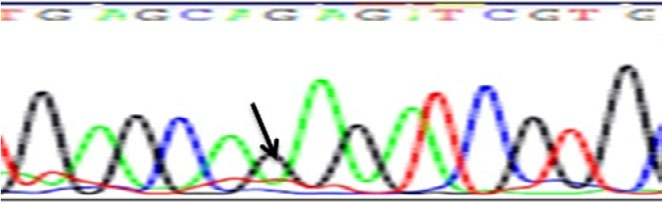
Electrophoretograms showing *GATA4* polymorphism rs56166237 (NM_002052.4:c.99 G/T) identified in homozygous samples (GG)

According to *GATA4* genotypes, a 5‐prime UTR variant rs368418329, GT (42% of the cases) and GG (46% of the cases) genotypes showed the most frequent presentation in cases (24 ASD, 48 VSD, six PDA, six aortic coarctation and 12 Fallot) (Table 5). While regarding a synonymous variant rs56166237, GT and GG were the most presented in cases (41.4%, 56% respectively) in contrast to the control group (20%, 1.7% respectively) (Figure 2). GT genotype was present in 15 ASD,18 VSD, three PDA, three aortic coarctation and nine Fallot's tetralogy .

**Table 5:** Comparison of cardiac data according to *GATA4* (rs368418329 and rs56166237) genotypes in all studied cases

*GATA4* (rs368418329)genotypes in all studied cases								
	GG *N* = 69		GT *N* = 63		TT *N* = 18		P1*	*P2#*
	*N*	%	*N*	%	*N*	%		
ASD	15	21.7	9	14.2*	3	16.7*#	0.00	0.02
VSD	27	39	21	33*	3	16.7*#	0.00	0.00
PDA	3	4.3	3	4.7	3	16.7*#	0.00	0.00
Aortic coarctation	0	0	6	9.5*	0	0*#*	0.00	0.00
Fallot tetralogy	6	8.6	6	9.5	0	0*#	0.00	0.00
	Mean	*SD*	Mean	*SD*	Mean	*SD*		
EF	55	9	58	10	60	6	>0.05	>0.05

*GATA4* (rs56166237) genotypes in all studied cases								
	GG *N* = 84		GT *N* = 62		TT *N* = 4		*P1*	*P2*
	*N*	%	*N*	%	*N*	%		
ASD	10	12	15	24*	2	50*#	0.00	0.00
VSD	31	37	18	29	2	50*#	0.00	0.00
PDA	6	7	3	4.8	0	0*#*	>0.05	0.00
Aortic coarctation	3	3.5	3	4.8	0	0*#*	>0.00	0.00
Fallot tetralogy	3	3.5	9	14.5*	0	0*#*	0.00	0.00
	Mean	*SD*	Mean	*SD*	Mean	*SD*		
EF	58	9	61	10.2	56	5	>0.05	>0.05

Note

GenBank reference sequence: variant rs368418329 in *GATA4* (NM_002052.4:c.‐294G/T) and synonymous variant rs56166237 in *GATA4* (NM_002052.4:c.99 G/T).

CT genotype of *TBX5* ‐rs6489957 was found in 12 ASD, 24 VSD, six PDA, three aortic coarctation and nine fallot that represent 42% of cases, while CC genotype present in 58% of the cases in contrast to 0.03% of control group (Table 6).

**Table 6:** Comparison of cardiac data according to *TBX5* (rs6489957) genotypes in all studied cases

	CC *N* = 87		CT *N* = 58		*P*
	*N*	%	*N*	%	
ASD	15	17	12	20	0.01
VSD	27	31	24	41	0.005
PDA	3	3.4	6	10.3	0.00
Aortic coarctation	3	3.4	3	6	>0.05
Fallot tetralogy	3	3.4	9	15.5	0.00
	Mean	*SD*	Mean	*SD*	
EF	57	0.11	55	0.13	>0.05

Note

GenBank reference sequence: variant rs6489957 in *TBX5* to sequence number NM_000192.3 (NM_000192.3:c.1281C/T).

## 4 DISCUSSION

There are key genes known to be crucial for cardiac growth. For example, *GATA4, NKX2‐5,* and *TBX5* may work in a complex manner to control a subgroup of genes required for cardiac septal formation (Rajagopal et al., 2007). Due to the discrepancy of results among diverse populations, we aimed at studying such link in Egyptian cohort having congenital heart diseases (CHDs). The study was intended to explain the relationship of five polymorphisms of *NKX2‐5, GATA4* and *TBX5* with susceptibility of having CHDs in Egyptian children.

Previous study found in the tissues of heart and blood, one recognized single‐nucleotide polymorphism (SNP) (rs2277923) in *NKX2‐5* and one recognized SNP (rs56166237) in *GATA4*. In contrast to our study, their study presented that *NKX2‐5* and *GATA4* mutations have no role in sporadic CHD pathogenesis (Yin et al., 2018).

Another author revealed seven mutations, (three in the intronic region, three in the coding region and one in 3′ UTR). Of the previous mutations, two (rs2277923 and a new mutation, D16N) were powerfully related to VSD. They establish that *NKX2‐5‐GATA4* complex, in case of mutant, had significant conformational alterations and harm of key polar interactions, which might be a reason of the pathogenic behaviour. D16N pathogenic mutation of *NKX2‐5* renders the structural–functional discrepancy that perhaps may lead to the diseased state (Mattapally, Singh, Murthy, Asthana, & Banerjee, 2018).

A previous study identified 53 mutations in the diseased cardiac tissues by direct sequencing, involving 35 nonsynonymous, 13 synonymous, 3′‐UTR and two intronic mutations. Three mutations (Arg25Cys, Thr178Met and Ala219Val) are recognized, also detected NCBI dbSNPs rs2277923 (A239G, Glu21Glu) was found in patients. Only heterozygous genotypes were attained. They obtained the genotypic frequency 22 AA: 17 AG: 6 GG for dbSNP rs2277923 in 45 samples of lymphocytic DNA (Reamon‐Buettner et al., 2004).

A new heterozygous DNA sequence variant (DSV), 1433A>G, was recognized in one tetralogy of Fallot (TOF) patient and one persistent left superior vena cava (PLSVC) patient, but none in controls. In agreement with our results, the occurrence of rs2277923 in CHD group was significantly increased than the control group. The allele and genotype were associated with the existence of CHD (Cao et al., 2015).

Amplicon libraries for 16 CHD‐strictly related genes were produced and sequenced from 68 CHD patients. Fourteen variants were existing in community databases with very rare allele incidence. One variant (p.Arg25Cys in *NKX2‐5*,) has been formerly related to CHD (Pulignani et al., 2018).

Three nonsynonymous variants in *NKX2‐5* were recognized in the heterozygous state by sequence investigation: p.Glu21Gln was established in single ASD‐II patient; and three patients had the p.Arg25Cys (R25C) variant. Contrary to our results, the p.Glu21Gln was also recognized in 0.88% of the controls. Their findings also support that variant of *NKX2‐5* is a polymorphism, as it was not significantly altered among DS patients with CHD and controls (Alcántara‐Ortigoza et al., 2015).

Nine new and 19 possibly pathogenic variants were established using Sanger sequencing. Analyses completed by sex discovered dissimilar variants related to bicuspid aortic valve (BAV) in men (*EGFR* rs533525993 and *TEX26* rs12857479) and females (*NKX2‐5* rs2277923) (Dargis et al., 2016).

The two complete coding exon and partial flanking intron sequences of *NKX2‐5* gene were screened using DNA sequencing in 107 ASD patients and 391 VSD patients as well as 487 healthy individuals (control) from the Yunnan area in China. The results showed that single–nucleotide polymorphism (rs2277923) was identified. The incidence of nucleotide polymorphism (rs2277923) was significantly greater in the ASD group, and the allele and genotype were related to the occurrence of ASD. rs2277923 SNP may contribute to the danger of sporadic ASD in Chinese people (Cao et al., 2016).

The previously stated variant (rs2277923) was present at significantly greater levels in the CHD population than in the control group. *NKX2‐5* mutations may be mosaic in nature, therefore deserving investigation in both blood and tissue samples (Ketharnathan, Koshy, Sethuratnam, Paul, & Venkatesan, 2015).

The authors examined mutations of the *NKX2‐5* coding region in 230 nonsyndromic Chinese Han CHD patients using denaturing HPLC and sequencing. Two recognized single–nucleotide polymorphisms (rs2277923 and rs3729753) were identified, contrary to our results, there were insignificant differences in the allele and genotype frequencies between CHD and the controls (*p* > 0.05) (Zhang et al., 2009).

Altered heterozygous *CSX/NKX2‐5* mutations were established in congenital heart defects patients in an autosomal dominant fashion. All missense mutations in the Home domain had decreased DNA binding and slight transcriptional role. While their study does not describe the genotype–phenotype association of the ten human mutations, they identify specific deformities of *CSX/NKX2‐5* function important for transactivation of target genes (Kasahara et al., 2000).

Previous study investigated the incidence and prevalence of *GATA4, NKX2‐5,* gene mutations in a large group of individuals with controtruncal defect (CTD), including 178 patients with tetralogy of Fallot, 13 patients with double–outlet right ventricle (DORV), and 11 patients with truncus arteriosus. The mutation study identified one recognized missense variant (Arg25Cys) in the *NKX2‐5* in two (1.1%) sporadic patients with TOF. These sequence alterations were absent in 500 matched controls (De Luca et al., 2011).

Heterozygous mutations in transcription factor gene *NKX2‐5* are connected to either isolated or combined congenital heart disease (CHD), primarily secundum atrial septal defect‐II (ASD‐II), ventricular septal defect (VSD) or tetralogy of Fallot (TOF). Importantly, sporadic cases of CHD that share phenotypic features of *NKX2‐5* mutation carriers were negative on genetic investigation. Thus, even significant for cardiac development, germline mutations in *NKX2‐5* are infrequent in patients with sporadic CHD and genetic heterogeneity is likely for sporadic forms of CHD (Stallmeyer, Fenge, Nowak‐Göttl, & Schulze‐Bahr, 2010).

Twelve distinct mutations in the *NKX2‐5* coding region were identified in 18 of 608 patients (3%) including 9 of 201 with Fallot tetralogy, 3 of 71 with ASD secundum, one with truncus arteriosus, one with double–outlet right ventricle. In one out of nine patients with the aortic and mitral valve and a small VSD identified heterozygosity for a C to T transition at nucleotide 73 (c.73C>T)of the *NKX2‐5* sequence. The identified nucleotide transition led to the substitution of an arginine at amino acid 25 to a cysteine (p.R25C). They did not find this gene variation in the parents or in the other 120 patients of the study and also in a control group of 380 healthy Caucasian (McElhinney, Geiger, Blinder, Benson, & Goldmuntz, 2003).

They found one formerly known *NKX2‐5* missense variation, heterozygous c.73C>T (p.Arg25Cys), in a 10‐year‐old child with Fallot tetralogy. The same heterozygous alteration was found also in two unrelated persons in the healthy control group. The study shows the presence of p.Arg25Cys in healthy control children other than African Americans, and in vitro study suggested a structural/functional change in the altered protein region (Akcaboy et al., 2008).

Four heterozygous mutations were recognized in six unrelated TOF patients, comprising three with pulmonary atresia and five with right aortic arch; none had ECG evidence of PR interval prolongation. Three of four mutations (Arg216Cys, Glu21Gln and Ala219Val) changed highly–conserved amino acids, of which two mapped in the conserved NK2 domain. The fourth mutation (Arg25Cys) was identified in three unrelated patients and has been previously reported (Goldmuntz, Geiger, & Benson, 2001).

Previous study recognized two diverse mutations in the *NKX2‐5* coding region among the 159 (1.26%) individuals. An Arg25Cys mutation was recognized in a Tetralogy of Fallot patient (Soheili et al., 2016). Moreover, a single–nucleotide polymorphism c.99G>T was detected in *GATA4*. Though, the polymorphic frequency in ASD cases was like that in healthy controls (for genotype GT, *p* = 0.3847; for allele T, *p* = 0.3950) (Liu et al., 2010).

## 5 CONCLUSION

In addition to reporting different genotype frequencies of two polymorphisms of *NKX2‐5* (rs2277923, rs28936670) in CHD cases, two variants in *GATA4* (rs368418329, rs56166237) and one synonomous variant in *TBX5* (rs6489957) seem to have a role in the pathogenesis of CHD. Our results propose the importance of numerous gene variants investigation of CHD children in Egypt.
